# Efficacy and Safety of Halometasone Cream to Treat Chronic Generalized Eczema and the Effects of Halometasone Cream on Serum Cortisol Levels

**DOI:** 10.1155/2017/3265024

**Published:** 2017-11-09

**Authors:** Yan Li, Wei Xu, Linfeng Li

**Affiliations:** Department of Dermatology, Beijing Friendship Hospital, Capital Medical University, Beijing 100050, China

## Abstract

The aim of the study was to investigate the efficacy and safety of halometasone cream to treat chronic generalized eczema and the effects of halometasone cream on serum cortisol (COR) levels. Sixty consecutive outpatients diagnosed with chronic generalized eczema between January and April 2017 were included and divided into groups A, B, and C with a lesion area of 30%–40%, 41%–50%, and 51%–60%, respectively. Groups A, B, and C were treated with halometasone cream with a daily dose of 15 g, 20 g, and 30 g for 7–14 days, respectively. Ten patients were randomly selected from each group for serum COR measurement at days 0, 7, and 14. On day 14, group B had significantly higher cure rate (47.1%) than groups A (17.9%) and C (13.3%) and significantly higher effectiveness rate (82.4%) than group C (40.0%) (all *P* < 0.05). Serum COR levels were not affected in group A but were reduced significantly in groups B and C on days 7 and 14 (all *P* < 0.05). No adverse reaction was observed. Halometasone cream appeared to relieve chronic generalized eczema effectively and safely. High dosage (≥20 g daily for 14 days) may temporarily reduce endogenous COR production substantially, although it may be more effective.

## 1. Introduction

Eczema is a skin inflammatory disease and can be caused by multiple intrinsic and extrinsic factors. Chronic generalized eczema, a common type of eczema, is characterized by lesions occurring at multiple sites, complex etiology, persistent recurrence, and severe pruritus. The disease substantially reduces the quality of life of patients and imposes serious economic burden on patients [1]. Topical glucocorticoid (GC), which is commonly used to treat eczema in clinical practice, can usually relieve eczema-associated symptoms such as inflammation and pruritus rapidly [2]. Topical GC use at a proper dosage is quite safe and barely causes adverse reactions [3]. However, patients with chronic generalized eczema often use topical GC for a long time and at a relatively high dose. Wester and colleagues have found that long-term use of topical GC may cause GC to enter circulation system through skin absorption, resulting in reversible hypothalamic-pituitary-adrenal axis suppression and the consequent systemic adverse reactions [4]. Coureau and colleagues have demonstrated that the weekly dose of topical propionic acid chloride betamethasone cream should be less than 50 g in order to prevent GC-associated severe adverse reactions [5].

Halometasone cream is a commonly used high-potency topical GC. It inhibits inflammation, epidermal hyperplasia, and allergic reactions, constrict blood vessels, and relieve pruritus. The mechanism of action of halometasone is that the drug can bind to steroid receptors to modulate the protein synthesis that is involved in the development of chronic generalized eczema and thus to regulate the function of inflammatory cells and lysosomes and ultimately to reduce inflammatory responses [6–8]. According to the instruction of halometasone cream, only 1.41% of halometasone cream used for consecutive 7 days on a lesion area of 400 cm^2^ can be absorbed via the skin. However, the dosage of halometasone cream use in the real world is often higher than the recommended dosage provided by the manufacturer. The effects of topical halometasone cream on serum cortisol (COR) levels remain unknown.

The current study aims to evaluate the efficacy and safety of halometasone cream to treat chronic generalized eczema and examine the effects of halometasone cream on serum COR levels. This study also provides clinical evidence for an effective and safe dosage of halometasone cream to treat the disease.

## 2. Materials and Methods

### 2.1. Study Design

This prospective cohort study included consecutive outpatients visiting the Department of Dermatology of Beijing Friendship Hospital for chronic generalized eczema from January to April 2017. The study protocol has been approved by the Institutional Review Board of Beijing Friendship Hospital (Approval number 2017-P2-010-02). All of the participating patients signed the informed consent form.

### 2.2. Patient Inclusion and Exclusion Criteria

The patient inclusion criteria were as follows: (1) a confirmed diagnosis of chronic generalized eczema according to the diagnostic criteria [10]; (2) the disease severity being at least moderate (Investigator's Global Assessment Score ≥ 3); (3) the skin lesions being at multiple sites and affected 30% to 60% of the total body surface area; (4) the target lesions being at the limbs or trunk and showing a diameter of 2 cm to 10 cm; (5) adult patients aged 18 to 80 years; (6) voluntarily signing the informed consent form.

The exclusion criteria were as follows: (1) skin lesions accompanied with infection of viruses, bacteria, and/or fungi; (2) the eczema on the face and skin-folding areas; (3) systemic use of glucocorticoid and/or immunosuppressant and/or ultraviolet light therapy within 4 weeks before the enrollment interview; (4) local use of glucocorticoid and/or oral use of antihistamines within two weeks before the enrollment interview; (5) severe disease in the liver, kidney, and/or blood; (6) autoimmune diseases, chronic serious infection, diabetes, mental diseases, substance abuse, and/or alcoholic; (7) the malignant tumor or other serious diseases that may compromise the accuracy of efficacy evaluation for the study drug; (8) any other condition that the investigators believed could interfere in the study; (9) pregnant, breastfeeding, or planning to become pregnant during the study period; (10) being allergic to the study drugs; (11) failing to follow the study protocol.

### 2.3. Intervention

Patients received the topical treatment of halometasone cream (trade name Aoneng, Hongkong Bright Future Pharmaceutical, Ltd., batch number: 1601508) twice daily. After the cream was applied, the treated area was massaged for 5 minutes until the cream was absorbed completely. For patients with a lesion area of 30%–40% (Group A), 41%–50% (Group B), and 51%–60% (Group C), the daily dose of the halometasone cream was 15 g, 20 g, and 30 g, respectively. The treatment course was 7 days. Patients were followed up every 7 days and the efficacy of the halometasone cream was evaluated. The treatment was ended on day 7 when the skin lesions were cured; otherwise, the treatment was continued for another 7 days. Adverse events were recorded during each follow-up examination. Clinical observation was ended on day 14 for all of the participating patients.

### 2.4. Serum Cortisol (COR) Measurement

Ten patients were randomly selected from each group for serum cortisol (COR) measurement on days 0, 7, and 14. Three mL venous blood was withdrawn at 8 a.m. before breakfast on days 0, 7, and 14. Patients with abnormal serum COR after the treatment with halometasone cream were required to come back for serum COR measurement 28 days after the termination of the treatment (day 42 in total). Serum COR levels were determined using the UniCel DxI 800 chemiluminescence immunoassay system (Beckman Coulter, USA) following the manufacturer's instruction. All reagents were provided by the manufacturer (Beckman Coulter, USA). The normal range of serum COR levels was 6.0–23.0 *μ*g/dL.

### 2.5. Halometasone Cream Efficacy Assessment

At the enrollment interview and each follow-up examination, the severity, shape, and area of skin lesions were determined. The shape of skin lesions was defined as erythema (E), induration/papulation (I), excoriation (Ex), and lichenification (L). The severity score was 0, 1, 2, or 3 representing none, light, moderate, or severe, respectively. Lesion area score was 0, 1, 2, 3, 4, 5, or 6 representing that 0%, < 10%, 10%–29%, 30%–49%, 50%–69%, 70%–89%, or 90%–100% of the each body region area were affected by the skin lesions, respectively. Eczema area and severity index (EASI) was calculated according to the following equation: head/neck lesion area score × total head/neck severity score (E + I + Ex + L) × 0.1 + upper limb lesion area score × total upper limb lesion severity score (E + I + Ex + L) × 0.2 + trunk lesion area score × total trunk lesion severity score (E + I + Ex + L) × 0.3 + lower limb lesion area score × total lower limb lesion severity score (E + I + Ex + L) × 0.4 [9]; efficacy index = (EASI before treatment − EASI after treatment) ÷ EASI before treatment × 100%. Completely effective, significantly effective, effective, and not effective were defined as an efficacy index > 90%, 60%–89%, 20%–59%, and <20%, respectively. Effectiveness rate = (the number of cases showing completely effective + the number of cases showing significantly effective) ÷ total number of cases × 100% [11].

### 2.6. Halometasone Cream Safety Assessment

Adverse reactions to halometasone cream were monitored during the study. Adverse reactions were discovered by telephone interviews and in-person follow-up examinations. The severity of adverse reactions was graded as mild, moderate, and severe. Treatments and outcomes of adverse reactions were recorded.

### 2.7. Statistical Analyses

The statistical analysis software SPSS 16.0 was used to analyze data. Numerical data are presented as mean ± standard deviation (SD). Intragroup comparison was analyzed by paired *t*-test and repeated measures ANOVA. Categorical data are presented as rate and compared by Chi-square test. *P* value was two-sided, and *P* < 0.05 was considered statistically significant.

## 3. Results

### 3.1. Patient Baseline Characteristics

**Figure 1:**
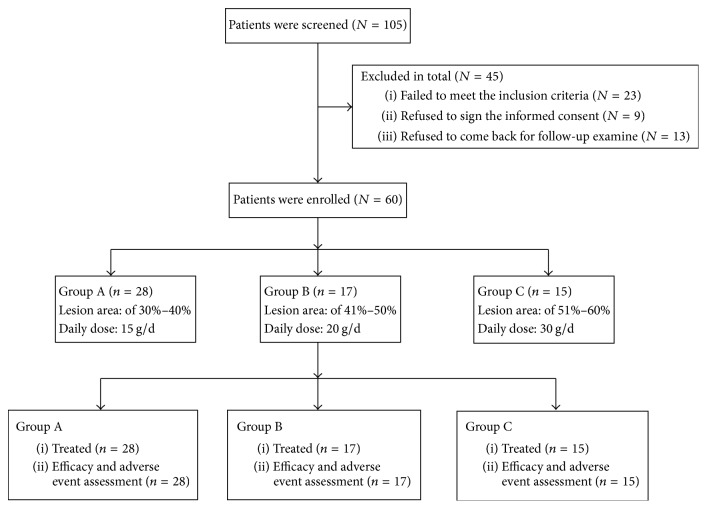
Patient flowchart.

A total of 60 patients (37 men and 23 women) with chronic generalized eczema were enrolled in the study. Patient flowchart is shown in Figure 1. Patients' baseline characteristics are displayed in Table 1. The 60 patients had a broad range of age (22–78 years), disease course (2–240 months), and skin lesion area (30%–60%). According to the skin lesion area, the 60 patients were divided into three groups: group A (*n* = 28) with a lesion area of 30%–40%, group B (*n* = 17) with a lesion area of 41%–50%, and group C (*n* = 15) with a lesion area of 51%–60%. Notably, the proportion of men and EASI were significantly different in the three groups (*P* < 0.05, Table 1). The mean EASI of group C (39.8 ± 4.9) was the highest in the three groups (Table 1) and significantly higher than that of group B (34.0 ± 7.5, *P* = 0.004) and of group A (23.9 ± 4.2, *P* < 0.0001). Group B had significantly higher average EASI than group A (*P* < 0.0001). Although group C showed the longest mean disease history (80.7 ± 89.4 months), the difference in the three groups was not statistically significant. These data suggest that patients with larger lesion area appear to have more severe chronic generalized eczema.

### 3.2. Efficacy

The mean EASI was significantly different in the three groups at days 0, 7, and 14 of the treatment (all *P* < 0.001, Table 2). Group C had significantly higher mean EASI than group B and group A at day 7 (*P* = 0.022 versus group B; *P* < 0.0001 versus group A) and at day 14 (*P* = 0.001 versus group B, *P* < 0.0001 versus group A) (Table 2). The mean EASI was similar in groups A and B at days 7 and 14. When EASI was compared at different time points in the same group, EASI reduced significantly as the treatment continued in all three groups (all *P* < 0.0001, Table 2), indicating that the treatment may be effective.

**Figure 2:**
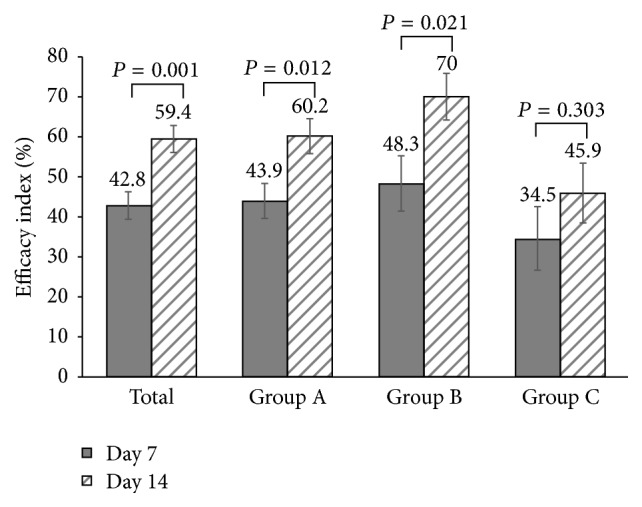
*Efficacy index at day 7 and day 14 of the treatment*. Intragroup comparison was analyzed by paired *t*-test. *P* value was 2-sided and *P* < 0.05 was considered significantly different.

Efficacy indexes at day 14 increased significantly compared with those at day 7 for total patient (*P* = 0.001), group A (*P* = 0.012), and group B (*P* = 0,021) but not for group C (Figure 2). Efficacy index was similar in the three groups at day 7. At day 14, the efficacy index of group B was the highest and significantly higher than that of group C (*P* = 0.009).

**Figure 3:**
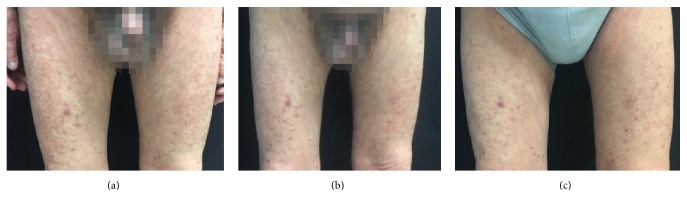
*Representative photos of lesions before and after treatment of a patient from group B*. (a) Lesion on day 0, EASI = 21.6. (b) Lesion on day 7, EASI = 9.6, efficacy index = 55.6%. (c) Lesion on day 14, EASI = 8.1, efficacy index = 62.5%.

After 7 days of topical treatment with halometasone cream, the cure rate and effectiveness rate of the total patients were 8.3% (5/60) and 35.0% (21/60), respectively (Table 3), and the two rates were not significantly different in the three groups (Table 3). The treatment was ended for patients showing completely effective (cured, *n* = 5), and the remaining 55 received the second 7-day treatment. At the day 14 follow-up visit, the cure rate and effectiveness rate in the 60 patients increased to 25.0% (15/60) and 63.3% (38/60), respectively (Table 3). Notably, the three groups showed significantly different cure rate (*P* = 0.044) and effectiveness rate (*P* = 0.046, Table 3). Group B had significantly higher cure rate (47.1%) than the group A (17.9%, *P* = 0.036) and group C (13.3%, *P* = 0.04) (Table 3). Effectiveness rate was significantly higher in group B (82.4%) than in group C (40.0%, *P* = 0.014, Table 3) at day 14 follow-up visit. Representative photos showing the skin lesions before and after the treatment of a patients from group B are displayed in Figure 3.

### 3.3. Serum COR Levels

**Figure 4:**
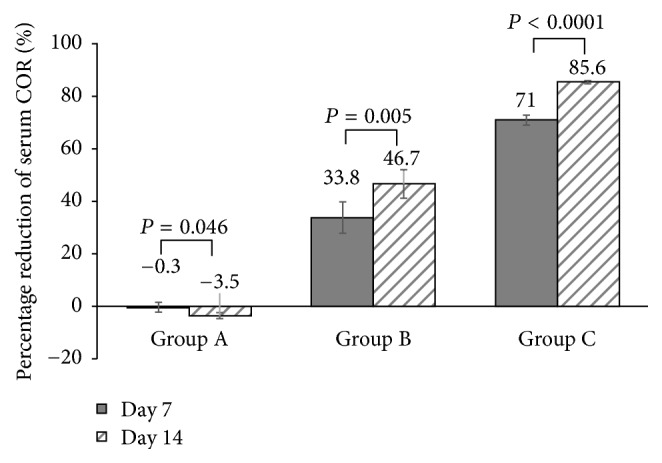
*Percentage reduction in serum COR levels*. Intragroup comparison was analyzed by paired *t*-test. *P* value was 2-sided and* P* < 0.05 was considered significantly different.

Ten patients were randomly selected from each group for serum COR measurement. At the enrollment (day 0), serum COR levels were similar in the three groups and in the normal range, whereas they were significantly different in the three groups at day 7 and day 14 (all *P* < 0.0001, Table 4). In group A, the serum COR remained at the similar level during the treatment, whereas the serum COR levels of both group B and group C reduced significantly at days 7 and 14 compared with the baseline values (all *P* < 0.0001, Table 4). At both day 7 and day 14, serum COR levels of group C were significantly lower than those of group A and group B (all *P* < 0.05), and group B also had significantly lower serum COR than group A (all *P* < 0.05). Similar to the absolute serum COR reduction during the treatment, the percentage reduction in serum COR was the greatest in group C and significantly higher in group C than in the other two groups at day 7 and day 14 (all *P* < 0.05, Figure 4). Patients with abnormally low serum COR after the treatment with halometasone cream came back for serum COR measurement 28 days after the termination of the treatment (day 42 in total). Their serum COR levels were restored to the normal levels.

No adverse reaction to halometasone cream was observed in the patients. All of the patients did not show any epidermal atrophy, skin irritation, hair follicle inflammation, or secondary bacterial, viral, or fungal infections on the lesion area.

## 4. Discussion

Halometasone cream is a potent topical GC drug and widely used in clinical practice. In the current study, the dosages of halometasone cream for the three patient groups were determined based on the previous report by Long and Finlay [12]. In their report, to guide dermatologists to prescribe a precise dosage of topical GC, the authors firstly proposed fingertip unit (FTU) [12]. A FTU is defined as the amount of topical GC cream that is from a standard 5 mm diameter topical medication tube and is enough to cover the area of the index finger from the fingertip to the nearest index finger joint [12]. One FTU is approximately equivalent to 0.5 g. The area of one hand accounts for approximately 1% of the total body surface area. One FTU (0.5 g) is for 2% of the total body surface area. Thus, one FTU per time for twice daily is equal to 1 g daily. The topical GC dosage used to treat bullous pemphigoid has been calculated according to lesion area and severity, and daily dose of topical propionic acid chloride betamethasone cream for mild, moderate, and severe bullous pemphigoid is 20 g, 30 g, and 40 g, respectively [13, 14]. Based on these previous reports [12–14], we determined the daily dosages for the three patient groups as 15 g for a lesion area of 30%–40%, 20 g for 41%–50%, and 30 g for 51%–60% in the current study.

This study found that the cure rate and effectiveness rate of one-week halometasone cream treatment for chronic generalized eczema were 8.3% and 35.0%, respectively, and the rates were increased to 25.0% and 63.3% as the treatment duration was increased to 2 weeks. Thus, halometasone cream alone appears to treat chronic generalized eczema rapidly and effectively. Previous studies have shown similar results [8, 15]. On day 14 of the treatment, group B showed significantly higher cure rate than the other two groups, suggesting that a daily dose of 20 g may be the optimal dose for patients with a lesion area of 41%–50%. Possible reasons for the lowest cure rate and effectiveness rate of group C may be associated with the relatively longer disease history and greater disease severity compared with groups A and B. In addition, in the current study, patients in group C were exposed to relatively high dosage of halometasone cream for two weeks, which could downregulate the GC receptors on epidermal keratinocytes and microvascular endothelial cells and consequently compromise the efficacy and anti-inflammatory effects of the topical GC [16]. Furthermore, the efficacy index of group C was not increased at day 14 compared with that at day 7, suggesting that daily 30 g of halometasone cream for two weeks may not be very effective for patients with severe chronic generalized eczema.

The serum COR levels of group A were not affected by the treatment. However, patients in the groups B and C showed significantly reduced serum COR levels after 7 and 14 days of the treatment. Serum COR levels in group C on day 14 of the treatment were even lower than the normal COR levels. In particular, after 14 days of treatment, the serum COR levels of group C were reduced by 85.6% compared with the baseline value. Topical use of GC can transiently increase serum COR levels, which triggers negative feedback to hypothalamus and anterior pituitary to downregulate endogenous COR production. Thus, long-term topical use of GC may actually reduce endogenous COR production. The dose and medication duration of topical GC may affect the extent of topical GC-associated serum COR reduction [17, 18]. In the current study, the treatment did not affect serum COR in group A, indicating that topical use of daily 15 g of halometasone cream for 14 days may not affect endogenous COR production in patients with a lesion area of 30%–40%. In contrast to the low dosage of halometasone cream, daily dose ≥ 20 g appeared to reduce endogenous COR production substantially. In particular, daily 30 g for only 7 days resulted in 71.0% decrease in serum COR levels. The high-dose halometasone cream-associated serum COR reduction was temporary. Serum COR levels were restored to normal levels 28 days after the termination of the treatment.

There are limitations in the current study. The sample size is relatively small. Only 10 patients in each group were measured for serum COR levels. Thus, the findings of the current study need to be verified by larger sale studies.

## 5. Conclusions

Topical use of low-dose halometasone cream alone appeared to relieve the symptoms of chronic generalized eczema effectively, rapidly, and safely. Daily 15 g for 14 days did not affect endogenous COR production. Although daily dose of 20 g for 14 days showed 82.4% effectiveness rate in patients with a lesion area of 41%–50%, serum COR levels of the patients were significantly reduced. Daily dose of 30 g for 7 days temporarily reduced serum COR to levels lower than the normal levels. Thus, we recommend avoiding high-dose halometasone cream to treat chronic generalized eczema.

## Figures and Tables

**Table 1:** Baseline characteristics.

	Total *N* = 60	Group A *N* = 28	Group B *N* = 17	Group C *N* = 15	*P* value
Age (year)					
Range (Min, Max)	22–78	23–78	32–74	22–78	
Mean ± SD	54.7 ± 16.1	54.8 ± 14.8	55.0 ± 14.0	54.1 ± 21.0	0.985

Sex					
Men, *n* (%)	37 (61.7)	15 (53.6)	15 (88.2)	7 (46.7)	0.026^*∗*^
Women, *n* (%)	23 (38.3)	13 (46.4)	2 (11.8)	8 (53.3)	

Lesion area (%)	30–60	30–40	41–50	51–60	
Disease history (month)					
Range (Min, Max)	2–240	3–240	6–120	2–240	
Mean ± SD	61.5 ± 57.6	55.8 ± 46.5	53.8 ± 33.7	80.7 ± 89.4	0.332

Lesion status					
EASI, Mean ± SD	30.7 ± 8.7	23.9 ± 4.2	34.0 ± 7.5^2^	39.8 ± 4.9^1^	<0.0001^*∗*^
Serum COR, Mean ± SD (*µ*g/dL)	10.9 ± 3.1	11.1 ± 4.3	10.9 ± 2.7	10.7 ± 2.0	0.967

SD: Standard deviation. EASI: eczema area and severity index. COR: Cortisol. One-way ANOVA was performed to compare the three groups. ^*∗*^*P* < 0.05 was considered significant different. ^1^Significantly higher versus group B (*P* = 0.004) and group A (*P* < 0.0001). ^2^Significantly higher versus group A (*P* < 0.0001).

**Table 2:** Patients' EASI before and after the halometasone cream treatment.

EASI Mean ± SD	Total *N* = 60	Group A *N* = 28	Group B *N* = 17	Group C *N* = 15	*P* value
At enrollment (Day 0)	30.7 ± 8.7	23.9 ± 4.2	33.9 ± 7.5	39.8 ± 4.9	<0.0001
Day 7 of the treatment	18.4 ± 11.6	14.0 ± 7.0	18.2 ± 11.9	27.0 ± 13.8^1^	0.001
Day 14 of the treatment	13.3 ± 10.8	9.9 ± 6.8	10.8 ± 10.1	22.3 ± 13.0^2^	<0.0001
*P* value	<0.0001	<0.0001	<0.0001	<0.0001	

EASI: eczema area and severity index. Repeated measures ANOVA was used to analyze the difference of the three groups. ^1^Significantly higher versus group B (*P* = 0.022) and group A (*P* < 0.0001). ^2^Significantly higher versus group B (*P* = 0.001) and group A (*P* < 0.0001).

**Table 3:** Halometasone cream efficacy.

	Total *N* = 60	Group A *N* = 28	Group B *N* = 17	Group C *N* = 15	*P* value
Follow-up visit at day 7					
Complete effective, *N* (%)	5 (8.3)	2 (7.1)	2 (11.8)	1 (6.7)	
Significant effective, *N* (%)	16 (26.7)	8 (28.6)	5 (29.4)	3 (20.0)	
Effective, *N* (%)	26 (43.3)	14 (50.0)	6 (35.3)	6 (40.0)	
No effect, *N* (%)	13 (21.7)	4 (14.3)	4 (23.5)	5 (33.3)	
Cure rate^1^ (%)	8.3	7.1	11.8	6.7	0.832
Effectiveness rate^2^ (%)	35.0	35.7	41.2	26.7	0.688

Follow-up visit at day 14					
Complete effective, *N* (%)	15 (25.0)	5 (17.9)	8 (47.1)	2 (13.3)	
Significant effective, *N* (%)	23 (38.3)	13 (46.4)	6 (35.3)	4 (26.7)	
Effective, *N* (%)	14 (23.3)	7 (25.0)	2 (11.8)	5 (33.3)	
No effect, *N* (%)	8 (13.3)	3 (10.7)	1 (5.9)	4 (26.7)	
Cure rate^1^ (%)	25.0	17.9	47.1^1^	13.3	0.044^*∗*^
Effectiveness rate^2^ (%)	63.3	64.3	82.4^2^	40.0	0.046^*∗*^

^1^Cure rate = the number of cases showing complete effective ÷ total number of cases. ^2^Effectiveness rate = (the number of cases showing complete effective + the number of cases showing significant effective) ÷ total number of cases × 100% [9]. Chi-square test was used to compare the difference of the three groups. ^*∗*^*P* < 0.05 was considered significant different. ^1^Significantly higher versus group A (*P* = 0.036) and group C (*P* = 0.04). ^2^Significantly higher versus group C (*P* = 0.014).

**Table 4:** Serum COR levels before and after the halometasone cream treatment.

Serum COR levels (*µ*g/dL)	Group A *N* = 10	Group B *N* = 10	Group C *N* = 10	*P* value
At enrollment (Day 0)	11.1 ± 4.3	10.9 ± 2.7	10.7 ± 2.0	0.967
Day 7 of the treatment	11.0 ± 3.9	6.9 ± 1.5^2^	3.0 ± 0.5^1^	<0.0001^*∗*^
Day 14 of the treatment	11.4 ± 4.2	5.5 ± 0.8^4^	1.65 ± 0.4^3^	<0.0001^*∗*^
*P* value	0.973	<0.0001	<0.0001	

COR: Cortisol. Repeated measures ANOVA was performed to analyze the difference. ^*∗*^*P* < 0.05 was considered significant different. ^1^Significantly lower versus group A (*P* < 0.0001) and group B (*P* = 0.001). ^2^Significantly lower versus group A (*P* = 0.001), ^3^significantly lower versus group A (*P* < 0.0001) and group B (*P* = 0.001). ^4^Significantly lower versus group A (*P* < 0.0001).
